# Supplementary material for: Case-Control Study on the Routes of Transmission of SARS-CoV-2 after the Third Pandemic Wave in Tuscany, Central Italy
Source: Int J Environ Res Public Health. 2023 Jan 20;20(3):1912. doi: 10.3390/ijerph20031912 (PMC9915363; doi:10.3390/ijerph20031912)
Supplement: Supplementary file 1 [file ijerph-20-01912-s001.zip › ijerph-2072258-supplementary.pdf]

Supplementary materials

Table S1. Summaries of the variables used in the analyses: variable name, variable explanation, number of missing values, frequency distribution calculated over the ten imputed datasets.

Variable name	Explanation	Number of missing values		Average proportions over the 10 imputations
AGE	What is your age group?	1	[0,19]	0.20
			[20,39]	0.30
			[40,59]	0.36
			>60	0.13
GENDER	What is your gender?	5	Male	0.37
			Female	0.63
VACCINE	Have you been vaccinated?	7	Yes	0.38
			No	0.62
MONTH	In which month have you been tested/was the swab carried out?	0	May	0.21
			June	0.20
			July	0.13
			August	0.21
			September	0.17
			October	0.08
ASL	To which Local Healthcare Authority do you belong?	18	Central	0.74
			North-West	0.15
			South-East	0.11
CITY SIZE	How many inhabitants live in your municipality of residence?	8	<5000	0.09
			[5,000-50,000)	0.50
			>50,000 or provincial capital	0.42
Test	What test result did you get?	46	Positive	0.80
			Negative	0.20
Outside region	Have you moved outside the Tuscany Region?	19	No	0.82
			Yes	0.18
Open-air restaurants	Have you dined at open-air restaurants?	25	No	0.49
			Yes	0.59
Indoor restaurants	Have you dined at indoor restaurants?		No	0.82
			Yes	0.18
Open air bars	Have you spent at least 15 minutes in a bar outdoors?	20	No	0.53
			Yes	0.47
Indoor bars	Have you spent at least 15 minutes in a bar indoors?	26	No	0.84
			Yes	0.16
Crowded clubs	Have you been in crowded clubs?	23	No	0.82
			Yes	0.18
Night clubs	Have you been to night clubs, e.g., discos, dance halls?	19	No	0.96
			Yes	0.04

Indoor sports	Have you performed physical activity in the presence of other people indoors (e.g., football, tennis, indoor swimming pools, gyms, etc.)?	20	No	0.93
			Yes	0.07
Outdoor sports	Have you carried out any team sport or physical activity in the presence of other people outdoors?	21	No	0.79
			Yes	0.21
Swimming-pool	Have you been in an outdoor pool?	99	No	0.92
			Yes	0.08
Seaside resort	Have you been at a seaside resort?	97	No	0.77
			Yes	0.23
Hands-washing	Have you washed your hands several time per day?	19	No	0.12
			Yes	0.88
Visiting Friends	Have you been visiting friends' or relatives' home?	19	No	0.35
			Yes	0.65
Hosting friends	Have you received friends or relatives at your home?	21	No	0.50
			Yes	0.50
Malls/supermarkets	Did you go to shopping malls or supermarkets to shop for food or other goods?	18	No	0.28
			Yes	0.72
Small stores	Have you gone to retail and neighbourhood stores to shop for food or other items?	19	No	0.47
			Yes	0.53
Indoor performances	Have you attended indoor shows or exhibitions?	20	No	0.92
			Yes	0.08
Outdoor performances	Have you attended outdoor shows or exhibitions?	23	No	0.89
			Yes	0.11
Hospitals	Have you been to a hospital/ambulatory/clinic/nursery home for medical appointments or to accompany someone?	19	No	0.71
			Yes	0.29
Libraries	Have you been to libraries or reading rooms?	30	No	0.97
			Yes	0.03
School/university	Have you been to school or university?	18	No	0.85
			Yes	0.15
Hair/Beauty salon	Have you been to the hairdresser, barber, beauty salon?	23	No	0.69
			Yes	0.31
Ceremonies	Have you participated in private parties, ceremonies, civil or religious festivals?	24	No	0.88
			Yes	0.12
Face mask	Have you made proper use of the face	22	No	0.14
			Yes	0.86

	mask when you were away from home?			
Job exposure	How much exposure to infection do you have at work?	33*	0: Low risk job (without a job/ working alone or outdoor)	0.51
			1: medium risk (jobs that imply contacts but not with patients)	0.36
			2: high risk (health sector with contact with patients)	0.13
Family size	How many people live with you?	5	0	0.10
			1	0.26
			2	0.25
			3	0.23
			4	0.11
			5	0.02
			6	0.01
			7	0.01
			8	0.00
			>9	<0.01
Public transport	Have you used public transport?	20*	0:no	0.82
			1: yes not crowded	0.10
			2: yes crowded	0.08
Courses	Have you attended classes or participated in the activities of associations/organisations/clubs (outdoor and indoor)?	22*	No	0.88
			Yes	0.12
Sporting events	Have you participated as a spectator in sporting events (outdoor and indoor)?	27*	No	0.94
			Yes	0.06
Religious services	Have you participated in religious services?	24	No	0.94
			Yes	0.06

Table S2. Number of respondents by exposure status and test result for each risk factor considered in the analysis*, and crude Odds Ratio (OR) calculated on the data in the previous columns.

	Unexposed		Exposed		
	Negative	Positive	Negative	Positive	Crude OR
Public transport 2	289,2	72,3	26,5	7,6	1,15
Public transport	289,2	72,3	36,2	8,2	0,91
Family size**	128	29,4	223,9	58,7	1,14
Job exposure 2	172,7	51,8	46,6	11,3	0,81
Job exposure	172,7	51,8	132,6	25	0,63
Face mask	42,1	18,5	309,8	69,6	0,51
Ceremionies	304,8	84,4	47,1	3,7	0,28
Hair/Beauty salon	229,5	73,5	122,4	14,6	0,37
Religious services	284,6	81,3	67,3	6,8	0,35
School/university	293,6	80,8	58,3	7,3	0,45
Libraries	341,5	87	10,4	1,1	0,42
Hospitals	235,8	74,6	116,1	13,5	0,37
Sporting events	330,4	83,4	21,5	4,7	0,87
Outdoor performances	307	85,7	44,9	2,4	0,19
Indoor performances	322	84,6	29,9	3,5	0,45
Small stores	152,6	55,1	199,3	33	0,46
Malls/supermarkets	88,7	33,4	263,2	54,7	0,55
Hosting friends	154,6	64,4	197,3	23,7	0,29
Visiting friends	101,1	52,6	250,8	35,5	0,27
Hand-washing	38,8	13,3	313,1	74,8	0,70
Courses	307,4	81,3	44,5	6,8	0,58
Seaside resort	272	67,2	79,9	20,9	1,06
Swimming-pool	323,6	79,8	28,3	8,3	1,19
Indoor sports	322,1	86	29,8	2,1	0,26
Outdoor sports	266,6	79,1	85,3	9	0,36
Night clubs	344,6	79,2	7,3	8,9	5,30
Crowded clubs	291,5	68,4	60,4	19,7	1,39
Indoor bars	290,3	79	61,6	9,1	0,54
Open air bars	190	44,9	161,9	43,2	1,13
Indoor restaurants	287,8	75,2	64,1	12,9	0,77
Open-air restaurants	167,4	46,5	184,5	41,6	0,81
Outside region	291,1	69,9	60,8	18,2	1,25

** Cell counts have been averaged over the 10 multiple imputed data sets, thus resulting in not integer numbers.*

***For the variable Family size, "Unexposed" corresponds to living alone or with only another person.*

Table S3. Results from the logistic regressions after excluding i) subjects with inconsistent answers about the reasons for testing, ii) subjects with missing test result. Estimate of the odds ratio for each risk factor, adjusted for the confounders, 90% confidence interval, and p-value.

	**Excluding subjects with inconsistent answers about the reason for testing**				**Excluding subjects with missing test result**			
	Estimate	LB 90%	UB 90%	p-value	Estimate	LB 90%	UB 90%	p-value
Ceremonies	0,225	0,077	0,662	0,023	0,216	0,072	0,647	0,022
Courses	0,536	0,237	1,211	0,210	0,617	0,272	1,399	0,333
Crowded clubs	1,601	0,892	2,874	0,187	1,393	0,759	2,554	0,371
Face mask	0,568	0,300	1,077	0,147	0,579	0,299	1,119	0,174
Family size	1,009	0,843	1,208	0,935	1,017	0,860	1,203	0,869
Hair/Beauty salon	0,487	0,257	0,923	0,065	0,496	0,264	0,932	0,068
Handwashing	0,827	0,411	1,665	0,657	0,972	0,469	2,014	0,949
Hospitals	0,502	0,273	0,923	0,063	0,519	0,282	0,956	0,078
Hosting friends	0,349	0,210	0,579	0,001	0,259	0,147	0,458	<0.001
Indoor bars	0,487	0,219	1,084	0,140	0,364	0,156	0,850	0,051
Indoor performances	0,393	0,089	1,731	0,302	0,102	0,017	0,627	0,039
Indoor restaurants	0,590	0,306	1,137	0,187	0,564	0,292	1,091	0,154
Indoor sports	0,407	0,197	0,841	0,042	0,365	0,169	0,788	0,032
Job exposure (1)	0,849	0,472	1,528	0,649	0,853	0,465	1,565	0,668
Job exposure (2)	1,755	0,731	4,212	0,292	2,062	0,873	4,873	0,168
Libraries	0,312	0,046	2,122	0,319	0,335	0,050	2,228	0,344
Malls/supermarkets	0,652	0,380	1,118	0,193	0,678	0,396	1,159	0,235
Night clubs	5,212	1,705	15,938	0,015	5,142	1,743	15,173	0,013
Open air bars	1,347	0,819	2,218	0,326	1,445	0,867	2,409	0,237
Open-air restaurants	0,981	0,600	1,605	0,950	1,069	0,648	1,762	0,828
Outdoor performances	0,182	0,049	0,677	0,033	0,142	0,038	0,532	0,015
Outdoor sports	0,219	0,058	0,825	0,06	0,289	0,079	1,064	0,118
Outside region	0,981	0,536	1,795	0,959	0,96	0,529	1,742	0,910
Public transport (1)	0,938	0,373	2,36	0,909	0,904	0,380	2,155	0,849
Public transport (2)	1,645	0,693	3,908	0,345	1,760	0,745	4,160	0,281
Religious services	0,474	0,211	1,062	0,129	0,492	0,220	1,099	0,148
School/university	0,496	0,208	1,184	0,186	0,446	0,176	1,129	0,154
Seaside resort	1,050	0,581	1,898	0,892	0,897	0,485	1,659	0,772
Small stores	0,580	0,362	0,93	0,058	0,525	0,318	0,867	0,035
Sporting events	1,164	0,402	3,364	0,815	1,141	0,389	3,346	0,841
Swimming-pool	0,898	0,357	2,255	0,847	0,819	0,340	1,975	0,710
Visiting friends	0,318	0,193	0,525	<0.001	0,246	0,145	0,418	<0.001

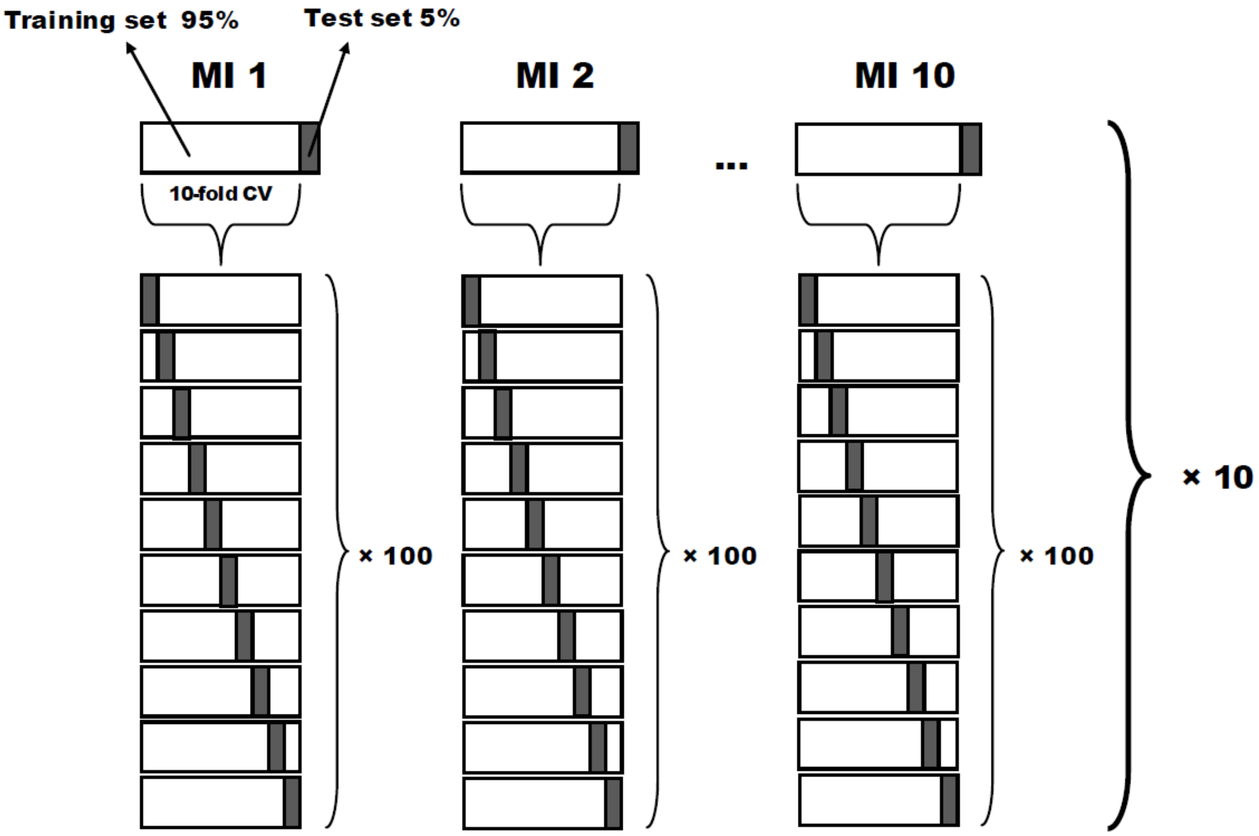

Figure S1. Cross-Validation scheme adopted to evaluate the predictive performance of the ridge logistic regression: The data set is randomly splitted in a training set (95% of units) and a test set (5%) (outer loop, repeated 10 times), and a 10-fold Cross-Validation is applied to the training set in order to select the penalty parameter (inner loop, repeated 100 times). The entire procedure is applied to each of the 10 imputed data sets and the average AUC is calculated.

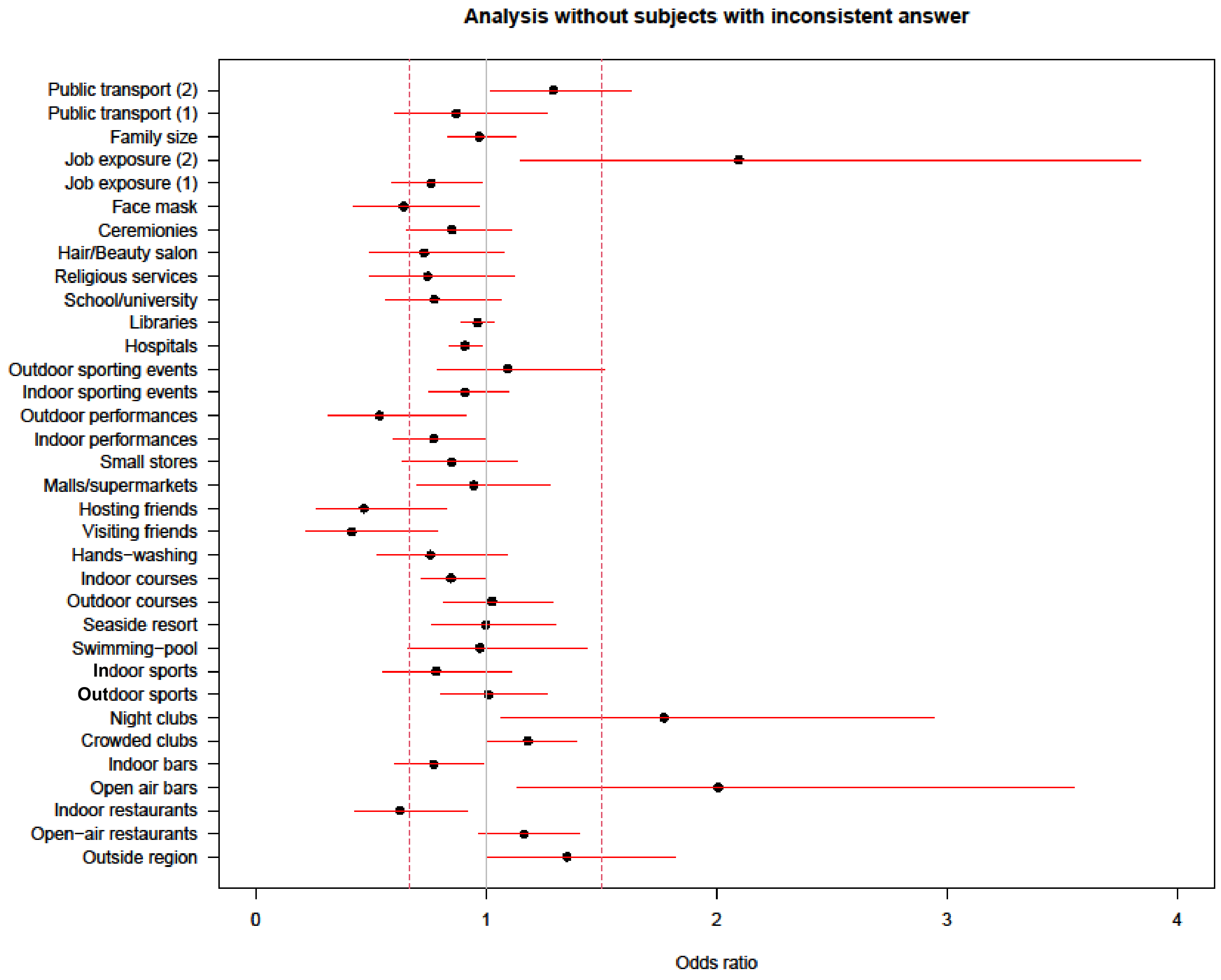

Figure S2. Results from the ridge regressions after excluding subjects with inconsistent answers about the reasons for testing. Estimate of the odds ratio of positivity for each risk factor, adjusted for all the other ones and for the confounders; the reported segments represent the 90% confidence intervals based only on the between-imputation variability.

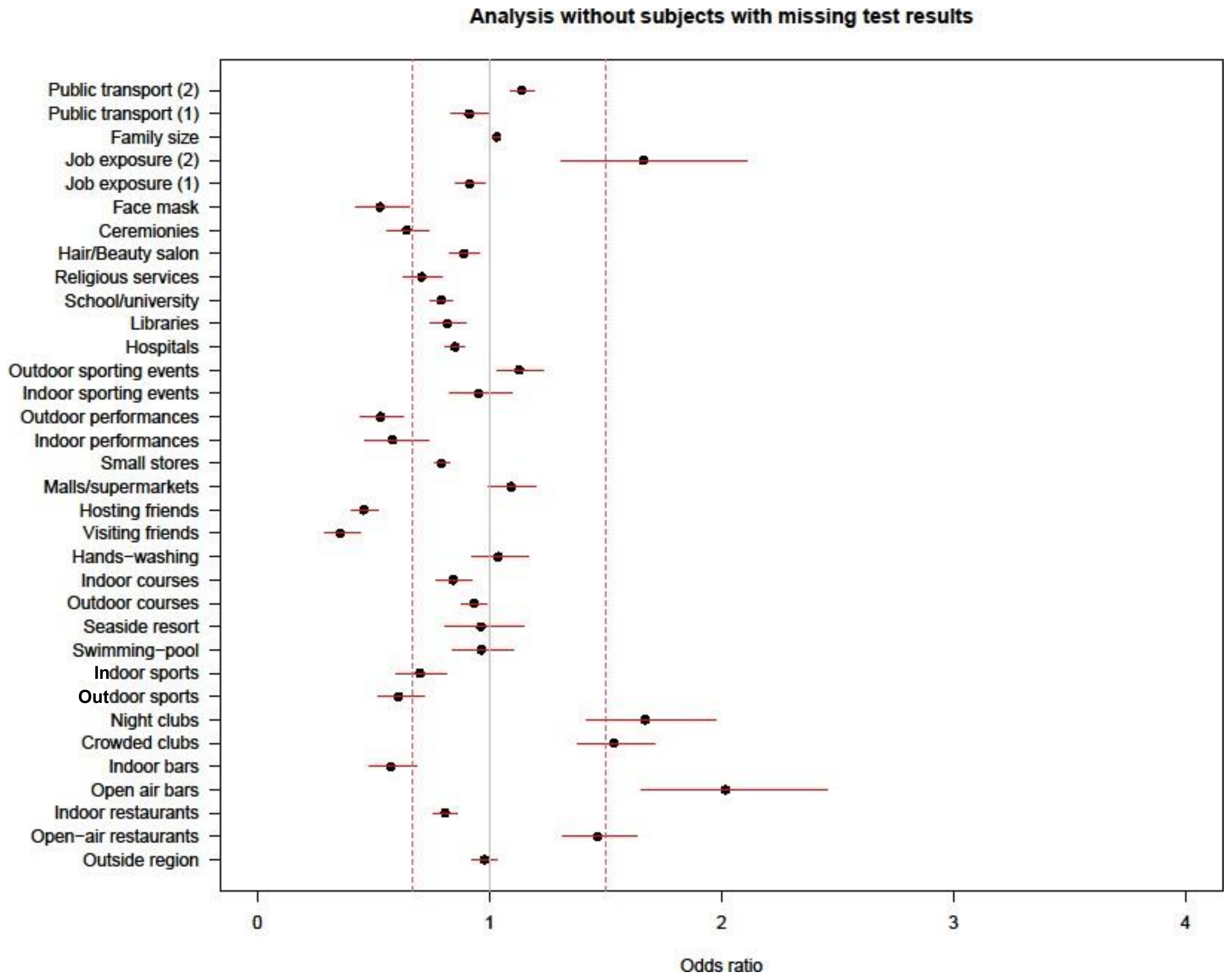

Figure S3. Results from the ridge regressions after excluding subjects with missing test result. Estimate of the odds ratio of positivity for each risk factor, adjusted for all the other ones and for the confounders; the reported segments represent the 90% confidence intervals based only on the between-imputation variability.

S1. The web-based questionnaire (translated from Italian)

1. What was the result of your recent Covid-19 test?

- ☐ Positive
- ☐ Negative

2. Why have you been tested for Covid-19? (It is possible to indicate more than one answer)

- ☐ Onset of symptoms
- ☐ Household contact with someone who tested positive for Covid-19
- ☐ Contact with someone who tested positive for Covid-19 other than household contacts
- ☐ Travel reasons
- ☐ Work reasons
- ☐ Pre-hospitalization
- ☐ Periodic testing (screening)
- ☐ As a precaution before visiting a family member or a friend (not household contact)
- ☐ Other

3. Was the swab performed to retest for COVID-19 after a positive test result to end isolation?

- ☐ Yes
- ☐ No

4. Has the Covid-19 test been prescribed by your family physician?

- ☐ Yes
- ☐ No

5. Where did you swab?

- ☐ At my local healthcare unit
- ☐ At a clinic affiliated with the National Healthcare System (e.g., Misericordia, Red Cross, etc.)
- ☐ At the family physician /pediatrician clinic
- ☐ In a drugstore
- ☐ Other

6. What kind of test did you take?

- ☐ A Nucleic Acid Amplification Tests (NAATs), such as PCR
- ☐ Antigenic test
- ☐ Unsure

7. Please indicate your age

- ☐ 0-9
- ☐ 10-13
- ☐ 14-19
- ☐ 20-29
- ☐ 30-39
- ☐ 40-49
- ☐ 50-59
- ☐ 60-69
- ☐ >69 years

8. What is your sex?

- ☐ Male
- ☐ Female
- ☐ Other

9. Are you a smoker?

- ☐ Yes
- ☐ No

10. How many people do you live with ?

Please select a number from 0 to 10 or >10

11. Please indicate the month the swab was carried out (from May to October)

11a. Please indicate in which decade of the month the swab was carried out

- ☐ 1-10
- ☐ 11-20
- ☐ 21-31

12. Please indicate the province of residence

- ☐ Arezzo
- ☐ Firenze
- ☐ Grosseto
- ☐ Livorno
- ☐ Lucca
- ☐ Massa-Carrara
- ☐ Pisa
- ☐ Pistoia
- ☐ Prato
- ☐ Siena

13. Please indicate the number of residents in your municipality

- ☐ < 5,000
- ☐ between 5,000 to 50,000
- ☐ > 50,000

14. Have you been vaccinated for COVID?

- ☐ Yes
- ☐ No

For those who answered "Yes" to question 14:

14a. Have you completed your scheduled vaccination course (two doses in the case of Pfizer, Moderna and AstraZeneca vaccines or 1 dose in the case of Johnson & Johnson vaccine)

- ☐ Yes
- ☐ No

14b. How long has it been since the last dose?

- ☐ <15 days
- ☐ 15 days - 1 month
- ☐ 1-3 months

From here on, answer the questions always referring to **<u>the 7 days prior to the onset of symptoms</u>** or, in case of no symptoms, **<u>to the 7 days prior to the date of the swab</u>**. Try to remember the best you can.

15. Has a household contact (i.e., family member or roommate) tested positive for COVID-19?

- ☐ Yes
- ☐ No

16. Have you used public transports?

- ☐ Yes
- ☐ No

For those who answered "Yes" to question 16:

16a. Please indicate the public transports you have taken

- ☐ City bus or tram
- ☐ Intercity Bus
- ☐ Taxi
- ☐ Underground
- ☐ Regional train
- ☐ High-speed train
- ☐ Airplane
- ☐ Other

16b. How often have you used public transports?

- ☐ Once or twice
- ☐ Occasionally
- ☐ Daily or almost daily

16c. How did you find the public transport you used?

- ☐ With few passengers
- ☐ With neither few nor too many passengers, but the respect of the distance of at least one meter from the others was guaranteed

☐ Crowded

17. Have you moved outside the Tuscany Region?

☐ Yes

☐ No

For those who answered "Yes" to question 17:

17a. Where have you been?

☐ Region of Northern Italy

☐ Another region of Central Italy

☐ Region of Southern Italy

☐ EU country

☐ Non-EU countries

18. Have you dined at a restaurant indoors?

☐ Yes, 1-2 times

☐ Yes, at 3 least times

☐ No

19. Have you dined at a restaurant outdoors?

☐ Yes, 1-2 times

☐ Yes, at 3 least times

☐ No

20. Have you spent at least 15 minutes in a bar indoors?

☐ Yes, 1-2 times

☐ Yes, at 3 least times

☐ No

21. Have you spent at least 15 minutes in a bar outdoors?

☐ Yes, 1-2 times

☐ Yes, at 3 least times

☐ No

22. Have you been to night clubs, e.g., discos, dance halls?

☐ Yes, 1-2 times

☐ Yes, at 3 least times

☐ No

23. Have you been in crowded spaces?

☐ Yes, 1-2 times

☐ Yes, at 3 least times

☐ No

24. Have you performed physical activity in the presence of other people indoors (e.g., football, tennis, indoor swimming pools, gyms, etc.)?

☐ Yes, 1-2 times

☐ Yes, at 3 least times

☐ No

25. Have you carried out any team sport or physical activity in the presence of other people outdoors?

☐ Yes, 1-2 times

☐ Yes, at 3 least times

☐ No

26. Have you been swimming in an outdoor pool?

☐ Yes

☐ No

27. Have you been to the beach?

☐ Yes

☐ No

28. Have you attended courses or participated in activities other than sports indoors (e.g., theater, music, singing, painting, drawing, writing, etc.)?

☐ Yes, 1-2 times

☐ Yes, at 3 least times

☐ No

29. Have you attended courses or participated in activities other than sports outdoors?

☐ Yes, 1-2 times

☐ Yes, at 3 least times

☐ No

30. Have you visited friends or family members at home?

☐ Yes, sometimes

☐ Yes, often

☐ No

31. Have you received visits from friends or family members at home?

☐ Yes, sometimes

☐ Yes, often

☐ No

32. Did you go to shopping malls or supermarkets to shop for food or other items?

☐ Yes, 1-2 times

☐ Yes, sometimes

☐ Yes, (almost) each day

☐ No

33. Have you gone to retail stores to shop for groceries or other items?

☐ Yes, 1-2 times

☐ Yes, sometimes

☐ Yes, (almost) each day

☐ No

34. Did you go to indoor shows or exhibitions (e.g., in cinemas, theaters, concert halls, museums, etc.)?

- ☐ Yes, 1-2 times
- ☐ Yes, at 3 least times
- ☐ No

35. Did you go to outdoor shows or exhibitions?

- ☐ Yes, 1-2 times
- ☐ Yes, at 3 least times
- ☐ No

36. Did you participate as a viewer in indoor sporting events?

- ☐ Yes, 1-2 times
- ☐ Yes, at 3 least times
- ☐ No

37. Did you participate as a viewer in outdoor sporting events?

- ☐ Yes, 1-2 times
- ☐ Yes, at 3 least times
- ☐ No

38. Have you been to a hospital/outpatient clinic/nursing care home for a medical examination or to accompany someone?

- ☐ Yes, 1-2 times
- ☐ Yes, at 3 least times
- ☐ No

39. Have you attended classes at school or at the university?

- ☐ Yes, 1-2 times
- ☐ Yes, occasionally
- ☐ Yes, (almost) each day
- ☐ No

40. Have you been to libraries or reading rooms?

- ☐ Yes, 1-2 times
- ☐ Yes, occasionally
- ☐ Yes, (almost) each day
- ☐ No

41. Have you attended religious services?

- ☐ Yes, 1-2 times
- ☐ Yes, at 3 least times
- ☐ No

42. Have you been to the hairdresser, barber shop, beauty center or similar?

- ☐ Yes
- ☐ No

43. Did you go to work outside your home?

- ☐ Yes, I never worked from home
- ☐ Yes, occasionally
- ☐ No, I always worked from home
- ☐ No, I have a job, but I have not been working in this period
- ☐ No, currently I do not have a job

For those who answered "Yes" to question 43:

43a. How many people have you had contact with at the workplace?

- ☐ With many people (20 or more)
- ☐ With a moderate number of people (less than 20 but more than five)
- ☐ With few people (five or less)
- ☐ I worked alone

43b. Where has your work mostly been done?

- ☐ Indoors
- ☐ Outdoors

44. Have you attended private parties, ceremonies, civil or religious celebrations (e.g., baptisms, weddings, birthday, graduation parties, etc.)?

☐ Yes

☐ No

45. Have you always used the mask correctly when you were away from home?

☐ Yes

☐ No

46. Have you sanitized your hands several times a day?

☐ Yes

☐ No

47. On a scale from 1 to 10, how would you rate your memory in answering the above survey questions, with 1 being "not at all reliable" and 10 "completely reliable"?

For those with a positive test result:

48. Where do you think you caught SARS-CoV-2? [select all that apply]

☐ From a household contact

☐ At work

☐ A school

☐ At the university

☐ In a nursing home

☐ At the hospital, at the doctor's clinic

☐ From non-cohabiting family members, friends, or acquaintances

☐ While carrying out sporting activities

☐ While carrying out cultural activities

☐ At a bar, restaurant, or pub

☐ In a shop/shopping mall

☐ At the cinema or theater

☐ Attending religious ceremonies or functions

☐ Traveling

☐ I don't know

☐ Other

49. Which of the following best describes your occupation? (It is possible to indicate only one answer)

- ☐ Entrepreneur, senior manager, legislator (e.g., entrepreneurs of large and small companies, directors and general managers, deans and rectors, prefects, governors)
- ☐ Intellectual, scientific, and highly specialized profession (e.g., engineers, architects, geologists, lawyers, judges, doctors, professors and teachers, researchers, analysts and designers of software and / or websites and / or systems)
- ☐ Technical profession (e.g., laboratory technician, nurse, midwife, programmer, expert, social worker, agents and sales representatives, sports instructors and coaches, air commanders and pilots, police officers)
- ☐ Executive professions in office work (e.g., office clerk, office secretary, post office or bank clerk, switchboard operator)
- ☐ Artisan, skilled worker, and farmer (e.g., bricklayers, mechanics, painters, electricians, livestock breeder)
- ☐ Plant operators, workers of fixed and mobile machinery and vehicle drivers (e.g., train driver, bus driver, bulldozer, blast furnace operator)
- ☐ Armed forces
- ☐ Other (e.g., cleaner, janitor, maid, laborer)

50. Do you have a job in the health sector?

- ☐ Yes
- ☐ No

For those who answered "Yes" to question 50
50a. Do you have contact with patients?

- ☐ Yes
- ☐ No
